# Clinical outcomes of aortic stenosis patients undergoing Impella-supported high-risk percutaneous coronary intervention

**DOI:** 10.3389/fcvm.2025.1638259

**Published:** 2026-01-07

**Authors:** Poonam Velagapudi, Lavanya Bellumkonda, David J. Cohen, Alexandra J. Lansky, Arsalan Abu-Much, Julia B. Thompson, Michael J. Schonning, Bjorn Redfors, Zhipeng Zhou, Cindy L. Grines, Aneel S. Maini, Yanru Li, Wayne B. Batchelor, William W. O’Neill

**Affiliations:** 1 Division of Cardiology, University of Nebraska Medical Center, Omaha, NE, United States; 2 Division of Cardiology, Yale School of Medicine, New Haven, CT, United States; 3 Clinical Trials Center, Cardiovascular Research Foundation, New York, NY, United States; 4 St. Francis Hospital, Roslyn, NY, United States; 5 Barts Heart Centre, London and Queen Mary University of London, London, United Kingdom; 6 Health Sciences, Weill Cornell Medicine, New York, NY, United States; 7 Department of Molecular and Clinical Medicine, Gothenburg University, Gothenburg, Sweden; 8 Department of Cardiology, Sahlgrenska University Hospital, Gothenburg, Sweden; 9 Department of Cardiology, Northside Hospital Heart Institute, Atlanta, GA, United States; 10 Division of Cardiology, University of Texas Southwestern, Dallas, TX, United States; 11 Inova Center of Outcomes Research, Inova Heart and Vascular Institute, Falls Church, VA, United States; 12 Center for Structural Heart Disease, Division of Cardiology, Henry Ford Health System, Detroit, MI, United States

**Keywords:** high-risk percutaneous coronary intervention, aortic stenosis, mechanical circulatory support, major adverse cardiovascular and cerebrovascular events, PROTECT III

## Abstract

**Background:**

Severe aortic stenosis (AS) is associated with an increased risk of adverse outcomes in patients undergoing percutaneous coronary intervention (PCI). While Impella-supported high-risk PCI (HRPCI) has demonstrated improved outcomes, its safety in patients with AS remains inadequately established.

**Objectives:**

We evaluated the effectiveness and safety of Impella support in patients with AS undergoing HRPCI.

**Methods:**

Patients from the PROTECT III study (NCT04136392), a single-arm, FDA-audited, multicenter investigation of Impella-supported HRPCI, were assessed. AS severity was classified as none/trivial, mild, moderate, or severe. The primary outcome was 90-day major adverse cardiac and cerebrovascular events [MACCE, defined as the composite of all-cause mortality, MI, stroke/transient ischemic attack (TIA), and revascularization]. Secondary outcomes included in-hospital complications, stroke/TIA, and vascular complications requiring surgery.

**Results:**

Of the 594 patients with available echocardiographic data, 490 had none/trivial AS, while 34, 24, and 46 had mild, moderate, or severe AS, respectively. Patients with AS were older, had fewer incidences of diabetes, were more likely to have left main disease, and had higher left ventricular ejection fraction. Severely calcified lesions and atherectomy were more frequent among patients with moderate or severe AS. No significant differences were observed in PCI-related complications, stroke/TIA, and 30-day or 90-day MACCE across AS severity groups. However, transfusion rates were higher in patients with AS.

**Conclusion:**

In patients undergoing Impella-supported HRPCI, 90-day MACCE and PCI-related complications were similar across all levels of AS severity. These findings suggest that the procedure is safe in this complex, high-risk population.

**Clinical Trial Information:**

*Trial Name*: The Global cVAD Study (cVAD). ClinicalTrial.gov URL: https://clinicaltrials.gov/ct2/show/NCT04136392?term=cvad&draw=2&rank=2. ClinicalTrial.gov Identifier: NCT04136392.

## Introduction

Aortic stenosis (AS) and coronary artery disease (CAD) frequently coexist because they share common pathophysiology mechanisms and risk factors (1, 2). More than half of the patients with AS who undergo aortic valve replacement (AVR) have concomitant CAD. In randomized controlled trials (RCTs) comparing surgical aortic valve replacement (SAVR) and transcatheter aortic valve replacement (TAVR) for severe AS, the reported prevalence of CAD ranged from 15% to 80% (3). In the United States, approximately 1 million percutaneous coronary interventions (PCIs) are performed annually (4), and about 10% of patients with severe AS undergoing TAVR require PCI either before, during, or after the procedure (5). Severe AS is associated with adverse outcomes in patients undergoing PCI (6), and this risk is further increased by comorbid conditions such as a depressed left ventricular ejection fraction (LVEF) or more complex CAD, including left main disease and heavy calcification.

The use of mechanical circulatory support with Impella has been shown to improve outcomes in patients undergoing high-risk percutaneous coronary intervention (HRPCI) (4, 7). However, severe AS, defined as an aortic valve area (AVA) ≤0.6 cm^2^, is considered a contraindication to Impella insertion. Although several reports describe successful Impella use for HRPCI in patients with severe AS, with or without aortic valve modification prior to insertion (8–11), patients with severe AS have been excluded from most clinical trials of Impella-assisted HRPCI (4, 12, 13). Therefore, we aimed to evaluate the safety and effectiveness of Impella-supported HRPCI across different degrees of AS severity.

## Methods

### Study design and oversight

Data from the PROTECT III study have been previously published (14, 15). Briefly, the PROTECT III study is an FDA-approved single-arm, prospective, observational study that enrolled 1,237 patients between March 2017 and March 2020 who underwent Impella-supported HRPCI at 46 centers across the United States of America. It is a substudy of the global cVAD registry (NCT04136392), which includes multiple postapproval studies intended to evaluate the safety and efficacy of Impella mechanical circulatory support across a variety of cardiovascular indications, such as acute myocardial infarction (MI) with cardiogenic shock, postcardiotomy cardiogenic shock, acute right ventricular dysfunction, and HRPCI without cardiogenic shock (16). The index HRPCI and postprocedural care were performed according to the discretion of the treating physician. Patients who required Impella as a rescue measure were not eligible for enrollment in PROTECT III.

In-hospital data on baseline characteristics, preprocedural echocardiography, procedural techniques, and clinical outcomes were collected from admission through discharge. Patients were also followed for the occurrence of clinical outcomes through 90 days and for their vital status through 1 year. Angiographic data were analyzed by the Beth Israel Deaconess Medical Center Angiographic Core Laboratory, and an independent clinical events committee adjudicated major adverse cardiac and cerebrovascular events (MACCE) through 90 days.

The study was conducted in accordance with the Declaration of Helsinki, and the protocol was approved by the applicable Institutional Review Board or Independent Ethics Committee at each participating center prior to patient enrollment. Patients were eligible for enrollment upon receiving an Impella device or at the time of attempted Impella device implantation and were considered enrolled in the study once a patient identification number was assigned in the study database. Medical history and in-hospital data were collected through a retrospective chart review under a waiver of informed consent. Informed consent for postdischarge follow-up was then obtained from surviving patients during the index hospitalization or within 40 days after discharge. An independent 12-member steering committee, comprising interventional cardiologists, cardiac surgeons, and heart failure specialists, facilitated the conduct of the cVAD study. The sponsor (Abiomed Inc., Danvers, MA, USA) supervised study management and source document verification and provided funding to the Cardiovascular Research Foundation (New York, NY, USA) for statistical analysis. The authors had unrestricted access to the study data and accept full responsibility for the integrity of this report. Artificial intelligence was not utilized in any stage of data curation, data analysis, or manuscript preparation. Data from this study may be available to support additional research; such requests should be directed to the corresponding author.

### Study population

Patients enrolled in the PROTECT III study were included in this analysis only if information on AS severity was available. AS severity data came from two sources: (1) site-reported information, where the participating sites provided details about AS severity categorized as none/trivial AS, moderate AS, and severe AS; and (2) echocardiography core laboratory data, which included multiple parameters related to the evaluation of AS severity, such as the aortic valve peak velocity (V_max_), aortic valve area, and aortic valve mean pressure gradient (ΔP_mean_). In cases where patient data were available from both sources, the echocardiographic core laboratory evaluation was used to determine the severity of AS. As the echocardiography core laboratory did not provide an adjudication for AS severity, the AS severity of the core laboratory data was assessed using an integrative approach based on the 2020 ACC/AHA Guideline for the Management of Patients with Valvular Heart Disease (17). Specifically, patients with V_max_ ≥ 4.0 m/s were categorized as having severe AS, patients with 3.0 m/s ≤ V_max_ < 4.0 m/s were categorized as having moderate AS, patients with 2.5 m/s ≤ V_max_ < 3.0 m/s were categorized as having mild AS, and patients with V_max_ < 2.5 m/s were categorized as having none or trivial AS. In cases where discrepancies existed between V_max_ and AVA (*N* = 8; 3.0 m/s ≤ V_max_ < 4.0 m/s and AVA ≤ 1.0 cm^2^), other parameters were employed to refine AS severity classification. Specifically, if LVEF was ≥50% and stroke volume index (SVI) was <35 mL/m^2^ (group D3; paradoxical low-flow severe AS), the patients were classified as having severe AS. If LVEF was ≥50% and SVI was ≥35 mL/m^2^, the patients were classified as having moderate AS. Patients with LVEF <50% (group D2; severe low-flow, low-gradient AS with reduced LVEF) were excluded from the analysis because further investigation would be required to confirm AS severity.

### Study endpoints

The primary outcome of the study was the rate of major adverse cardiac and cerebrovascular events (MACCE) at 90 days, defined as the composite of all-cause mortality, MI, stroke/transient ischemic attack (TIA), and repeat revascularization.

The secondary outcomes included (1) in-hospital PCI related complications, defined as a composite of no reflow, abrupt closure, dissection, intramural hematoma, distal embolus, side-branch occlusion, failure of stent deployment, stent thrombosis, stent jail, perforation, MI, arrhythmia, and cardiac arrest; (2) in-hospital stroke/TIA; and (3) in-hospital vascular complications requiring surgery.

### Statistical analysis

Baseline patient characteristics are presented as the mean ± standard deviation or median [Q1, Q3] for continuous measures and as proportions for categorical variables, where applicable. For time-to-first event analyses, event rates were estimated using the Kaplan–Meier method and compared with the log-rank test. Cox proportional hazards models were constructed for the primary composite outcome (90-day MACCE) and for 1-year all-cause mortality, adjusting for age, LVEF, and LM disease. All *p*-values are two-tailed, and *p* < 0.05 was considered significant for all analyses. All statistical analyses were performed using SAS version 9.4 (SAS Institute Inc., Cary, NC, USA).

## Results

### Baseline patient characteristics

Of the 1,237 patients enrolled in the PROTECT III study, 594 had pertinent echocardiographic data to diagnose AS and assess its severity. Of these, 36% were derived from echocardiographic core laboratory data and 64% were derived from site-reported data (Supplementary Figure S1). Among these patients, 490 (82.4%) had no/trivial AS, whereas 34 (5.7%), 24 (4.0%), and 46 (7.7%) patients had mild, moderate, and severe AS, respectively (Supplementary Figure S1). Baseline patient characteristics are summarized in Table 1. The median (IQR) age of the overall study population was 72 (63–81) years; 41% of patients were older than 75 years, 28.2% were women, and 69.3% were Caucasian. Patients with AS were older (mean ± SD): no/trivial AS (69.7 ± 11.0), mild AS (77.4 ± 8.9), moderate AS (79.8 ± 9.8), and severe AS (79.1 ± 10.3) (*p* < 0.0001). Baseline characteristics of patients included in this analysis vs. those excluded are presented in Supplementary Table S1. Compared to patients who had available echocardiography data, those without such data were more hypertensive (94.2% vs. 89.3%, *p* = 0.002), had a higher prevalence of dyslipidemia (82.6% vs. 77.1%, *p* = 0.02), exhibited a greater incidence of prior MI (43.5% vs. 37.0%, *p* = 0.02) and CAD (87.2% vs. 78.8%, *p* < 0.0001), and had lower LVEF (32.5 ± 14.7 vs. 35.3 ± 15.6, *p* = 0.007).

**Table 1:** Baseline characteristics.

Characteristic	No/trivial AS	Mild AS	Moderate AS	Severe AS	*P*-value
	(*N* = 490)	(*N* = 34)	(*N* = 24)	(*N* = 46)	
Demographics					
Age, years	69.7 ± 11.0	77.4 ± 8.9	79.8 ± 9.8	79.1 ± 10.3	<0.0001
Sex, male	71.8% (352/490)	82.4% (28/34)	70.8% (17/24)	63.0% (29/46)	0.31
Race	66.9% (328/490)	82.4% (28/34)	66.7% (16/24)	84.8% (39/46)	0.03
White/Caucasian	11.2% (55/490)	8.8% (3/34)	25.0% (6/24)	2.2% (1/46)	0.03
Black/African American	4.3% (21/490)	0% (0/34)	0% (0/24)	0% (0/46)	0.20
Asian	0.6% (3/490)	0% (0/34)	0% (0/24)	0% (0/46)	0.89
American Indian or Alaska Native	0.2% (1/490)	0% (0/34)	0% (0/24)	0% (0/46)	0.98
Native Hawaiian/Pacific Islander	4.3% (21/490)	2.9% (1/34)	0% (0/24)	2.2% (1/46)	0.65
Other	12.4% (61/490)	5.9% (2/34)	8.3% (2/24)	10.9% (5/46)	0.65
Unknown race	66.9% (328/490)	82.4% (28/34)	66.7% (16/24)	84.8% (39/46)	0.03
Body mass index, kg/m^2^	28.7 ± 6.6	29.1 ± 5.3	28.0 ± 4.8	27.0 ± 5.9	0.34
Medical history					
HTN	87.8% (425/484)	94.1% (32/34)	100% (24/24)	95.7% (44/46)	0.08
Dyslipidemia	76.0% (365/480)	82.4% (28/34)	83.3% (20/24)	80.4% (37/46)	0.65
History of tobacco use	61.7% (295/478)	81.3% (26/32)	54.5% (12/22)	71.1% (32/45)	0.08
Diabetes mellitus	60.7% (294/484)	55.9% (19/34)	41.7% (10/24)	34.8% (16/46)	0.003
Anemia	16.5% (72/436)	24.1% (7/29)	30.4% (7/23)	25.0% (11/44)	0.16
PVD	19.5% (94/482)	17.6% (6/34)	29.6% (7/24)	18.6% (8/43)	0.68
Chronic pulmonary disease	24.1% (115/478)	29.4% (10/34)	12.5% (3/24)	15.6% (7/45)	0.27
Prior stroke/TIA	17.0% (82/482)	21.2% (7/33)	29.2% (7/24)	15.6% (7/45)	0.43
CKD	28.1% (135/480)	36.4% (12/33)	33.3% (8/24)	37.8% (17/45)	0.42
eGFR^⸹^, mL/min/1.73 m^2^	69.9 ± 24.8	60.2 ± 24.3	62.1 ± 23.8	65.5 ± 22.2	0.09
On dialysis	32.6% (44/135)	8.3% (1/12)	62.5% (5/8)	29.4% (5/17)	0.09
Prior MI	39.3% (184/468)	37.5% (12/32)	25% (6/24)	18.6% (8/43)	0.03
CAD	77.7% (373/480)	85.3% (29/34)	83.3% (20/24)	82.6% (38/46)	0.59
Prior PCI	35.1% (170/484)	36.4% (12/33)	26.1% (6/23)	21.7% (10/46)	0.25
Prior CABG	12.2% (59/485)	11.8% (4/34)	4.2% (1/24)	0% (0/46)	0.055
Angina	40.3% (180/447)	48.3 (14/29)	42.9% (9/21)	26.7% (12/45)	0.24
CHF	57.6% (276/479)	67.6% (23/34)	60.9% (14/23)	58.7% (27/46)	0.71
Prior pacemaker/ICD/CRT implantation	16.3% (75/459)	13.3% (4/30)	13.0% (3/23)	10.9% (5/46)	0.75
AF	34.2% (13/38)	40% (2/5)	0% (0/1)	50% (1/2)	0.85
Admission characteristics					
Acute MI at the time of presentation	38.3% (176/460)	46.7% (14/30)	27.3% (6/22)	10.9% (5/46)	0.001
STEMI	10.6% (18/170)	0% (0/13)	16.7% (1/6)	0% (0/5)	0.22
NSTEMI	81.8% (139/170)	92.3% (12/13)	83.3% (5/6)	100% (5/5)	0.49
Unstable angina	7.6% (13/170)	7.7% (1/13)	0% (0/6)	0% (0/5)	0.82
Echocardiography characteristics					
VHD	13.2% (59/448)	20.0% (6/30)	65.2% (15/23)	75.6% (34/45)	<0.0001
LVEF, %	34.0 ± 14.9	41.0 ± 17.8	39.7 ± 19.0	42.3 ± 15.9	0.0002
Angiography characteristics					
LM disease	57.6% (281/488)	79.4% (27/34)	70.8% (17/24)	71.1% (32/45)	0.02
Number of diseased vessels					0.39
1	7.9% (38/483)	9.1% (3/33)	8.7% (2/23)	13.0% (6/46)	0.68
2	30.6% (148/483)	18.2% (6/33)	30.4% (7/23)	37.0% (17/46)	0.35
3	59.8% (289/483)	66.7% (22/33)	60.9% (14/23)	50% (23/46)	0.48
>3	1.7% (8/483)	6.1% (2/33)	0% (0/23)	0% (0/46)	0.18
Number of vessels treated					0.87
1	28.0% (129/460)	26.7% (8/30)	30.4% (7/23)	32.6% (15/46)	0.91
2	45.7% (210/460)	36.7% (11/30)	43.5% (10/23)	45.7% (21/46)	0.81
3	26.3% (121/460)	36.7% (11/30)	26.1% (6/23)	21.7% (10/46)	0.54
Number of lesions treated per patient	2.0 (2.0, 3.0)	2.0 (1.0, 3.0)	2.0 (1.0, 3.0)	2.0 (1.0, 3.0)	0.55

Values are mean ± standard deviation, median (Q1, Q3), or % (*n*/*N*). AF, atrial fibrillation; AS, aortic stenosis; CABG, coronary artery bypass graft; CAD, coronary artery disease; CHF, congestive heart failure; CIED, cardiac implantable electronic devices; CKD, chronic kidney disease; COPD, chronic obstructive pulmonary disease; CRT, cardiac resynchronization therapy; eGFR, estimated glomerular filtration rate; HTN, hypertension; LM, left main; LVEF, left ventricular ejection fraction; ICD, implantable cardioverter defibrillator; MI, myocardial infarction; NSTEMI, non ST-elevation myocardial infarction; PCI, percutaneous coronary intervention; PVD, peripheral vascular disease; STEMI, ST-elevation myocardial infarction; TIA, transient ischemic attack; VHD, valvular heart disease.

^⸹^eGFR calculated using the 2021 Chronic Kidney Disease Epidemiology Collaboration Creatinine equation.

Comorbid conditions such as hypertension, chronic kidney disease, peripheral vascular disease, prior stroke/TIA, and congestive heart failure were similar across groups (*p* ≥ 0.05). However, diabetes mellitus, prior MI, and left main (LM) disease differed significantly among patients with no/trivial AS, mild AS, moderate AS, and severe AS (Table 1). Patients with AS had a lower prevalence of diabetes mellitus (trivial/no AS, 60.7%; mild AS, 55.9%; moderate AS, 41.7%; severe AS, 34.8%; overall *p*-value = 0.003), a lower incidence of prior MI (trivial/no AS, 39.3%; mild AS, 37.5%; moderate AS, 25%; severe AS, 18.6%; overall *p*-value = 0.03), and a greater prevalence of LM disease (trivial/no AS, 57.6%; mild AS, 79.4%; moderate AS, 70.8%; severe AS, 71.1%; overall *p*-value = 0.02). Overall, LVEF was reduced and significantly differed between groups, with patients with mild, moderate, or severe AS demonstrating higher LVEF compared with those who had trivial or no AS (trivial/no AS, 34.0 ± 14.9; mild AS, 41.0 ± 17.8; moderate AS, 39.7 ± 19.0; severe AS, 42.3 ± 15.9; *p* = 0.0002). Patients with AS also had a higher prevalence of other valvular heart disease compared with those with no/trivial AS (no/ trivial AS, 13.2%; mild AS, 20.0%; moderate AS, 65.2%; severe AS, 75.6%; *p* < 0.0001). There were no significant differences in left ventricular (LV) and right ventricular (RV) dimensions, 2D LV end-diastolic/systolic volume, stroke volume index, and cardiac output index across AS severity groups. Additional echocardiographic features stratified by AS severity are presented in Supplementary Table S2.

### Angiographic and procedural characteristics

Angiographic and procedural patient characteristics stratified by AS severity are shown in Supplementary Tables S3 and S4, respectively. Femoral arterial access was used in 79.7% of patients with no/trivial AS, 80% with mild AS, 95.7% with moderate AS, and 93.5% with severe AS (*p* = 0.04). The median [IQR] number of lesions treated during the index cardiac catheterization procedure was similar across groups (no/trivial AS, 2.0 [2.0, 3.0]; mild AS, 2.0 [1.0, 3.0]; moderate AS, 2.0 [1.0, 3.0]; or severe AS, 2.0 [1.0, 3.0]; *p* = 0.55). Severe calcification was higher in the moderate AS group (no/trivial AS, 51.1%; mild AS, 50%; moderate AS, 71.1%; and severe AS, 59.3%; *p* = 0.03). The use of atherectomy was higher among patients with moderate or severe AS compared with no/trivial AS (no/trivial AS, 37.1%; mild AS, 51.5%; moderate AS, 75.0%; severe AS, 54.3%; *p* = 0.0002). PCI for LM was performed in 46.1% of patients with no/trivial AS, 61.8% with mild AS, 50% with moderate AS, and 58.7% with severe AS (*p* = 0.14). No significant differences were observed in the pre-PCI or post-PCI TIMI flow across groups.

Although pre-PCI ischemia jeopardy scores did not differ significantly across groups (trivial/no AS, 9.0 ± 2.1; mild AS, 9.8 ± 1.3; moderate AS, 9.1 ± 2.3; and severe AS, 8.5 ± 2.4; *p* = 0.10), post-PCI ischemia jeopardy scores did (trivial/no AS, 2.1 ± 2.2; mild AS, 1.5 ± 1.7; moderate AS, 2.0 ± 2.7; and severe AS, 1.1 ± 1.4; *p* = 0.02). Pre-PCI SYNTAX scores (no/trivial AS, 28.8 ± 12.8; mild AS, 31.0 ± 9.2; moderate AS, 32.6 ± 15.6; or severe AS, 21.8 ± 10.5; *p* = 0.009) and post-PCI SYNTAX scores (trivial/no AS, 7.5 ± 8.8; mild AS, 5.1 ± 6.6; moderate AS, 6.8 ± 9.8; or severe AS, 3.4 ± 4.3; *p* = 0.046) differed significantly across groups (Supplementary Table S3).

Contrast volume did not differ significantly among the no/trivial AS (207.1 ± 104.3 mL), mild AS (195.9 ± 120.4 mL), moderate AS (220.4 ± 150 mL), and severe AS (208.1 ± 111.5 mL) groups (*p* = 0.87). Supplementary Table S4 summarizes the Impella device-related procedural characteristics of this study. Impella CP was used in 66.7% of patients with no/trivial AS, 70.6% with mild AS, 95.8% with moderate AS, and 80.4% with severe AS (*p* = 0.007). Impella 2.5 was used in 33.3% of patients with no/trivial AS, 29.4% with mild AS, 4.2% with moderate AS, and 19.6% with severe AS (*p* = 0.007). Impella was successfully implanted in 99.7% of patients (592/594). Median Impella support duration differed significantly across groups (no/trivial AS, 4.64 ± 11.73 h; mild AS, 3.80 ± 8.63 h; moderate AS, 13.08 ± 42.07 h; and severe AS, 1.62 ± 1.25 h; *p* = 0.01).

### Clinical outcomes

#### Primary outcome

**Figure 1:**
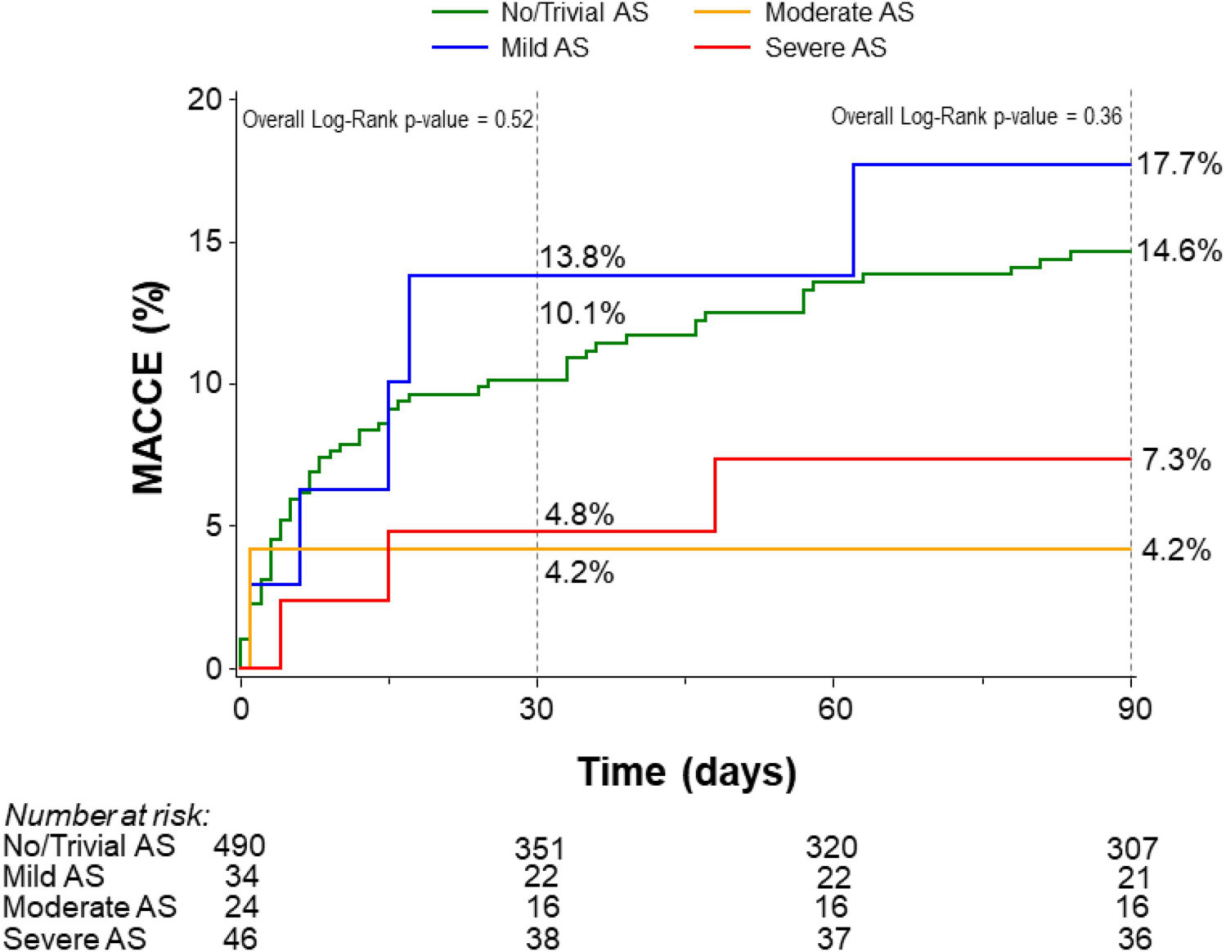
Kaplan–Meier curves for the rates of 30- and 90-day MACCE stratified by the degree of aortic stenosis. AS, aortic stenosis; MACCE, major adverse cardiovascular and cerebrovascular events.

The primary outcome, defined by the rate of 90-day MACCE using Kaplan–Meier analysis, did not differ significantly among patients with no/trivial AS (14.6%), mild AS (17.7%), moderate AS (4.2%), or severe AS (7.3%) (*p* = 0.36) (Figure 1). This finding remained consistent after adjustment for age, LVEF, and LM disease (*p* = 0.28). The rate of 90-day MACCE was similar between patients with severe AS and those with non-severe AS (7.3% vs. 14.4% respectively, *p* = 0.19) (Supplementary Figure S2). There were no significant differences in all-cause mortality, cardiovascular mortality, or non-cardiovascular mortality across AS severity categories (Table 2). A subgroup analysis comparing no/trivial/mild/moderate AS to severe AS also showed no significant difference in the rate of 90-day MACCE (14.4% vs. 7.3%, respectively, *p* = 0.20) (Supplementary Figure S2). Importantly, these results should be interpreted with caution because the study lacks sufficient power to detect differences due to the small sample size in the severe group.

**Table 2:** Rates of 30- and 90-day major adverse cardiac and cerebrovascular events and 1-year mortality stratified by the severity of aortic stenosis.

Endpoint	No/trivial AS	Mild AS	Moderate AS	Severe AS	*P*-value
30-day MACCE*	10.1% (44)	13.8% (4)	4.2% (1)	4.8% (2)	0.52
Death	8.4% (36)	10.8% (3)	4.8% (1)	4.8% (2)	0.76
Non-cardiovascular	0.7% (3)	0% (0)	0% (0)	0% (0)	0.88
Cardiovascular	7.7% (33)	10.8% (3)	4.8% (1)	4.8% (2)	0.81
MI	2.0% (8)	0% (0)	4.2% (1)	2.4% (1)	0.68
Stroke/TIA	1.7% (8)	2.9% (1)	0% (0)	0% (0)	0.66
Repeat revascularization	0.8% (3)	4.0% (1)	0% (0)	2.4% (1)	0.39
90-day MACCE*	14.6% (61)	17.7% (5)	4.2% (1)	7.3% (3)	0.36
Death	11.9% (49)	18.5% (5)	4.8% (1)	7.3% (3)	0.49
Non-cardiovascular	0.7% (3)	4.3% (1)	0% (0)	0% (0)	0.36
Cardiovascular	11.3% (46)	14.8% (4)	4.8% (1)	7.3% (3)	0.67
MI	4.1% (15)	0% (0)	4.2% (1)	2.4% (1)	0.71
Stroke/TIA	2.1% (9)	2.9% (1)	0% (0)	0% (0)	0.65
Repeat revascularization	2.2% (8)	4.0% (1)	0% (0)	2.4% (1)	0.85
1-year mortality	22.3% (85)	30.6% (8)	16.0% (3)	23.8% (9)	0.79

Event rates are Kaplan–Meier event rates, compared by the log-rank test.

AS, aortic stenosis; MACCE, major adverse cardiac and cerebrovascular events; MI, myocardial infarction; TIA, transient ischemic attack.

* MACCE is defined as the composite of all-cause death, myocardial infarction, stroke/TIA, and repeat revascularization.

#### Secondary outcomes

Table 3 presents the secondary outcomes of the study. In-hospital PCI-related complications were similar among patients with no/trivial AS (5.1%), mild AS (0.0%), moderate AS (0.0%), and severe AS (4.5%) (*p* = 0.42). There were no significant differences in stroke/TIA (no/trivial AS, 1.4%; mild AS, 0%; moderate AS, 0%; and severe AS, 0%; *p* = 0.68) or in vascular complications requiring surgery (trivial AS 1.4%, mild AS 0.0%, moderate AS 4.2%, and severe AS 0.0%, *p* = 0.36) during hospitalization across groups.

**Table 3:** Immediate PCI-related complications and in-hospital adverse events.

Event	No/trivial AS (*N* = 490)	Mild AS (*N* = 34)	Moderate AS (*N* = 24)	Severe AS (*N* = 46)	*P*-value
PCI-related complications^⸹^	5.1% (23/447)	0% (0/30)	0% (0/22)	4.5% (2/44)	0.42
No reflow	0% (0/447)	0% (0/30)	0% (0/22)	0% (0/44)	N/A
Abrupt closure	0.2% (1/447)	0% (0/30)	0% (0/22)	0% (0/44)	0.98
Dissection	0.7% (3/447)	0% (0/30)	0% (0/22)	2.4% (0/44)	0.89
Distal embolization	0.2% (1/447)	0% (0/30)	0% (0/22)	0% (0/44)	0.98
Perforation	1.8% (8/447)	0% (0/30)	0% (0/22)	0% (0/44)	0.63
Intramural hematoma	0.2% (1/447)	0% (0/30)	0% (0/22)	0% (0/44)	0.98
Side branch occlusion	0% (0/447)	0% (0/30)	0% (0/22)	0% (0/44)	N/A
Failure of stent deployment	0.4% (2/447)	0% (0/30)	0% (0/22)	2.3% (1/44)	0.43
Stent thrombosis	0% (0/447)	0% (0/30)	0% (0/22)	0% (0/44)	N/A
Stent jail	0.2% (1/447)	0% (0/30)	0% (0/22)	0% (0/44)	0.98
Myocardial ischemia	0% (0/447)	0% (0/30)	0% (0/22)	0% (0/44)	N/A
Myocardial infarction	0% (0/447)	0% (0/30)	0% (0/22)	0% (0/44)	N/A
Arrhythmia	0.2% (0/447)	0% (0/30)	0% (0/22)	0% (0/44)	0.98
Cardiac arrest	0% (0/447)	0% (0/30)	0% (0/22)	2.3% (0/44)	0.19
Adverse events					
Cardiac perforation	0.8% (4/490)	0% (0/34)	0% (0/24)	0% (0/46)	0.84
Pericardial effusion requiring pericardiocentesis	1.2% (6/490)	0% (0/34)	0% (0/24)	2.2% (1/46)	0.78
Cardiac arrest	2.4% (12/490)	0% (0/34)	4.2% (1/24)	4.3% (2/46)	0.62
Cardiogenic shock	1.8% (9/490)	0% (0/34)	4.2% (1/24)	2.2% (1/46)	0.71
Ventricular arrhythmia	2.7% (13/490)	0% (0/34)	0% (0/24)	0% (0/46)	0.42
Hypotension/hypotension during support	3.5% (17/490)	2.9% (1/34)	4.2% (1/24)	0% (0/46)	0.63
Severe heart failure requiring IV inotrope, ultrafiltration, or MCS	0.4% (2/490)	0% (0/34)	0% (1/24)	0% (0/46)	0.93
AKI (stage 2 or 3)	3.7% (18/490)	2.9% (1/34)	4.2% (1/24)	4.3% (2/46)	0.99
Life-threatening/disabling/major bleeding (BARC ≥3a)	2.0% (10/490)	2.9% (1/34)	4.2% (1/24)	0% (0/46)	0.65
Hemolysis	1.2% (6/490)	2.9% (1/34)	0% (0/24)	0% (0/46)	0.63
Thrombocytopenia	0.2% (1/490)	0% (0/34)	4.2% (1/24)	2.2% (1/46)	0.02
Anemia requiring transfusion	5.9% (29/490)	14.7% (5/34)	16.7% (4/24)	13.0% (6/46)	0.02
Vascular/cardiac structural complication requiring surgery/re-intervention	1.0% (5/490)	0% (0/34)	4.2% (1/24)	0% (0/46)	0.36
Neurologic dysfunction (stroke or TIA)	1.4% (7/490)	0% (0/34)	4.2% (1/24)	0% (0/46)	0.68
Limb ischemia	1.4% (7/490)	0% (0/34)	8.3% (2/24)	2.2% (1/46)	0.06
In-hospital mortality	4.5% (22/490)	5.9% (2/34)	4.2% (1/24)	6.5% (3/46)	0.92

Values are % (*n*/*N*) AKI, acute kidney injury; AS, aortic stenosis; BARC, bleeding academic research consortium; IV, intravenous; MCS, mechanical circulatory support; PCI, percutaneous coronary intervention; TIA, transient ischemic attack.

Kaplan–Meier analysis showed no significant differences in the rate of 30-day MACCE after hospital discharge among patients with trivial/no AS (10.1%), mild AS (13.8%), moderate AS (4.2%), and severe AS (4.8%) (*p* = 0.52) (Figure 1).

Other site-reported adverse events during discharge are presented in Table 2. Anemia requiring blood transfusion differed significantly among groups (trivial/no AS, 5.9%; mild AS, 14.7%; moderate AS, 16.7%; and severe AS, 13.0%; *p* = 0.02), with AS patients who had AS requiring more transfusions regardless of severity. There was no significant difference in severe heart failure requiring intravenous inotropes, ultrafiltration, or mechanical circulatory support (trivial/no, AS 0.4%; mild AS, 0%; moderate AS, 0%; and severe AS, 0%, *p* = 0.93) across groups.

#### Outcomes at 1 year

**Figure 2:**
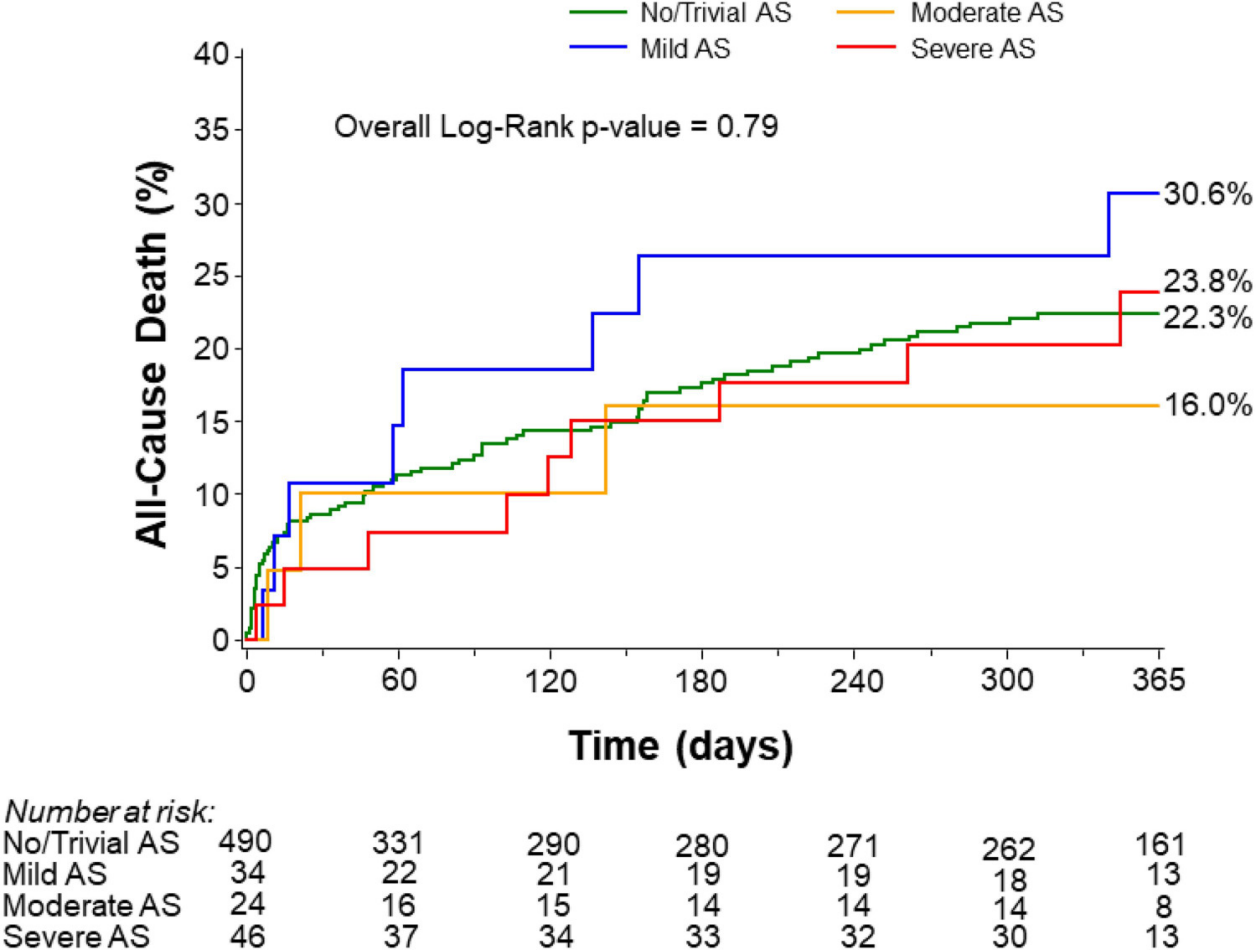
All-cause mortality at 1 year stratified by the degree of aortic stenosis. AS, aortic stenosis.

Kaplan–Meier analysis showed no significant differences in 1-year mortality among patients with trivial/no AS (22.3%), mild AS (30.6%), moderate AS (16.0%), and severe AS (23.8%) (Figure 2). These findings remained consistent after adjustment for age, LVEF, and LM disease (*p* = 0.76). The rate of 1-year mortality was similar between patients with severe AS and those with non-severe AS (23.8% vs. 22.6%, *p* = 0.91) (Supplementary Figure S3). As stated previously, although the rates appear similar, the study lacks sufficient power to detect differences.

## Discussion

The main findings of our study are as follows: Among patients in the PROTECT III trial who underwent Impella-assisted HRPCI and had echocardiographic data available to diagnose AS, we found that (1) there were no significant differences in the composite primary endpoint of 90-day MACCE across AS severity groups; (2) secondary outcomes, including PCI-related complications, stroke/TIA, or vascular complications requiring surgery based on AS status or severity, did not differ significantly by AS status or severity; and (3) 1-year mortality was similar regardless of AS status or severity. These results suggest that Impella use during HRPCI is safe and feasible in patients with AS.

Coronary artery disease (CAD) and AS frequently coexist due to shared common pathophysiology mechanisms and risk factors. Severe AS in patients undergoing PCI has been associated with an increased risk of adverse outcomes (6, 18). Mechanical circulatory support in patients undergoing HRPCI may improve procedural outcomes (7). Although the PROTECT II trial comparing intra-aortic balloon pump (IABP)-assisted HRPCI with Impella-assisted HRPCI found no significant differences in major adverse events (MAEs) between across groups at 30 days, there was a trend toward a lower incidence of MAEs at 90 days in the Impella-assisted HRPCI group in the intention-to-treat population (13). However, patients in the Impella arm had better hemodynamic support compared with those in the IABP group, as evidenced by the maximal drop in cardiac power output from baseline (−0.04 ± 0.24 vs. −0.14 ± 0.27, *p* = 0.001) (13). In a study comparing 504 PROTECT II-like patients enrolled in the PROTECT III trial with 216 patients from the Impella arm of the PROTECT II trial, the rate of 90-day MACCE was significantly lower in PROTECT III patients (15.1% vs. 21.9%, *p* = 0.037), accompanied by more complete revascularization and fewer bleeding events (4). The current ACC/AHA/SCAI coronary revascularization guidelines (19) provide a class IIb recommendation for the elective use of mechanical circulatory support during HRPCI.

The presence of severe AS is a relative contraindication to Impella use; therefore, patients with severe AS have generally been excluded from Impella-assisted HRPCI trials (4, 13). However, case reports and series have demonstrated the safety and feasibility of Impella use in patients with severe AS undergoing HRPCI (6, 9–11). In a recent single-center case series of 15 patients with severe AS, Yeo et al. reported that Impella insertion was safe and feasible for HRPCI before TAVR, without any associated mortality, stroke/TIA, or major vascular or bleeding complications (11). All but one patient underwent successful Impella insertion prior to HRPCI, and two patients required balloon aortic valvuloplasty (BAV) prior to Impella insertion (11). Importantly, 99.7% of all patients in the PROTECT III study—including those with AS—had a successful implantation of Impella for HRPCI, further confirming that the use of Impella in AS patients is feasible.

In patients with AS undergoing PCI, the risk and complexity of PCI may be further increased by coexisting conditions such as depressed LVEF and greater complexity of coronary disease, including LM disease, complex multivessel disease, and extensive coronary calcification. A study comparing the outcomes of patients who underwent PCI with and without severe AS showed that AS patients with severely reduced LVEF (<30%) or high STS scores (>10) experienced higher 30-day mortality compared with those with LVEF >30% or STS scores <10 (20). Among patients with severe AS undergoing TAVR who have complex LM or proximal CAD, PCI prior to TAVR carries a class IIa recommendation (17). Therefore, a substantial number of patients with severe AS and complex CAD undergo PCI prior to TAVR, and in such cases, mechanical circulatory support with Impella may be considered.

In our study, patients with AS had a higher prevalence of severe coronary calcification and LM disease, and the use of rotational atherectomy was also more common in this group. Despite this, PCI-related complications did not differ based on AS status or severity. Patients with AS undergoing TAVR typically require large-bore access for valve delivery using 14–16-Fr arterial sheaths. Similarly, Impella also requires a large-bore sheath (14 Fr) for insertion, making it essential to minimize vascular access complications to preserve the feasibility of future TAVR procedures. In our analysis, there was no significant difference in vascular complications requiring surgery based on AS status or severity. We found a slightly higher transfusion rate in patients with AS, which may be explained by the higher prevalence of anemia in this population. Approximately one-third of patients with moderate to severe AS have anemia (21). The etiology of anemia among patients with aortic stenosis is multifactorial. The most common causes include intravascular hemolysis caused by red blood cell fragmentation as high-velocity turbulent blood passes through the stenotic aortic valve (22) and gastrointestinal bleeding (Heyde's syndrome), which occurs due to an acquired coagulopathy (acquired von Willebrand syndrome 2A) (23). Patients with aortic stenosis are treated with antiplatelet and/or anticoagulant therapy, which further contributes to the risk of bleeding and the development of anemia.

Placement of the Impella device requires crossing the aortic valve to position it within the LV, theoretically increasing the risk of stroke due to embolization of calcium from the stenotic valve. However, our study found no significant difference in stroke/TIA either at discharge or at 1-year follow-up based on AS status or severity. Notably, there were no stroke/TIA events among patients with severe AS. These findings support the neurological safety of Impella use in patients with AS.

The PROTECT IV trial (ClinicalTrials.Gov NCT04763200) is an ongoing RCT designed to evaluate outcomes of Impella-assisted HRPCI compared with HRPCI without Impella support. However, similar to previous trials of Impella-assisted HRPCI, patients with severe AS are excluded from enrollment. In the absence of RCTs, our study represents the largest available analysis comparing outcomes of Impella-assisted HRPCI based on the presence and severity of AS. We observed no significant differences in the rates of 90-day MACCE or mortality based on AS status or severity in patients undergoing Impella-supported HRPCI. These findings are encouraging and hypothesis-generating.

In the future, an RCT comparing the outcomes of Impella-assisted HRPCI in patients with and without severe AS will be necessary to provide definitive answers regarding the safety and efficacy of Impella use in severe AS patients. Until such data are available, it remains essential to carefully select AS patients for HRPCI with Impella support, utilizing a multidisciplinary heart team approach and a shared decision-making process.

## Limitations

This study is a retrospective analysis of registry data and includes only patients with available echocardiographic data to confirm or refute the diagnosis of AS; therefore, it is subject to selection bias. The majority (64%) of echocardiographic data were from the participating sites without core laboratory adjudication, making them susceptible to interobserver variability. This study may not be representative of all patients with severe AS, as those with low-flow, low-gradient severe AS were likely excluded due to the lack of additional testing required to confirm the diagnosis. Some important clinical data—including changes in symptoms and NYHA class, heart failure hospitalizations, and echocardiographic measures, such as changes in LVEF over time—are not available. The relatively small number of patients with AS in the cohort limits the statistical power and generalizability of the findings, particularly for the subgroup of patients with severe AS. Nevertheless, the results of the study are reassuring and hypothesis-generating, providing a basis for the design of larger, prospective RCTs in this patient population.

## Conclusion

Among patients undergoing Impella-supported HRPCI, the rates of 90-day MACCE, in-hospital PCI-related complications, and 1-year mortality did not differ based on the presence or severity of AS.

## Data Availability

The original contributions presented in the study are included in the article/Supplementary Material; further inquiries can be directed to the corresponding author.
