# Supplementary material for: Clinical outcomes of aortic stenosis patients undergoing Impella-supported high-risk percutaneous coronary intervention
Source: Front Cardiovasc Med. 2026 Jan 7;12:1638259. doi: 10.3389/fcvm.2025.1638259 (PMC12819752; doi:10.3389/fcvm.2025.1638259)
Supplement: Supplementary file 1 [file Datasheet1.pdf]

Clinical Outcomes of Aortic Valve Stenosis Patients Undergoing Impella-Supported High-Risk Percutaneous Coronary Intervention

Poonam Velagapudi, MD; Lavanya Bellumkonda, MD; David J. Cohen, MD, MSc; Alexandra J. Lansky, MD; Arsalan Abu-Much, MD; Julia B. Thompson, MS; Michael J. Schonning, MS, MBS; Björn Redfors, MD, PhD; Zhipeng Zhou, MA; Cindy L. Grines, MD; Aneel Maini, MD; Yanru Li, MS, MPH; Wayne Batchelor, MD; William W. O'Neill, MD

Supplemental Material

**Supplemental Figure S1**	Page 2
**Supplemental Figure S2**	Page 3
**Supplemental Figure S3**	Page 4
**Supplemental Table S1**	Page 5
**Supplemental Table S2**	Page 6
**Supplemental Table S3**	Page 7
**Supplemental Table S4**	Page 8

Supplemental Figure S1: Study Flow Chart

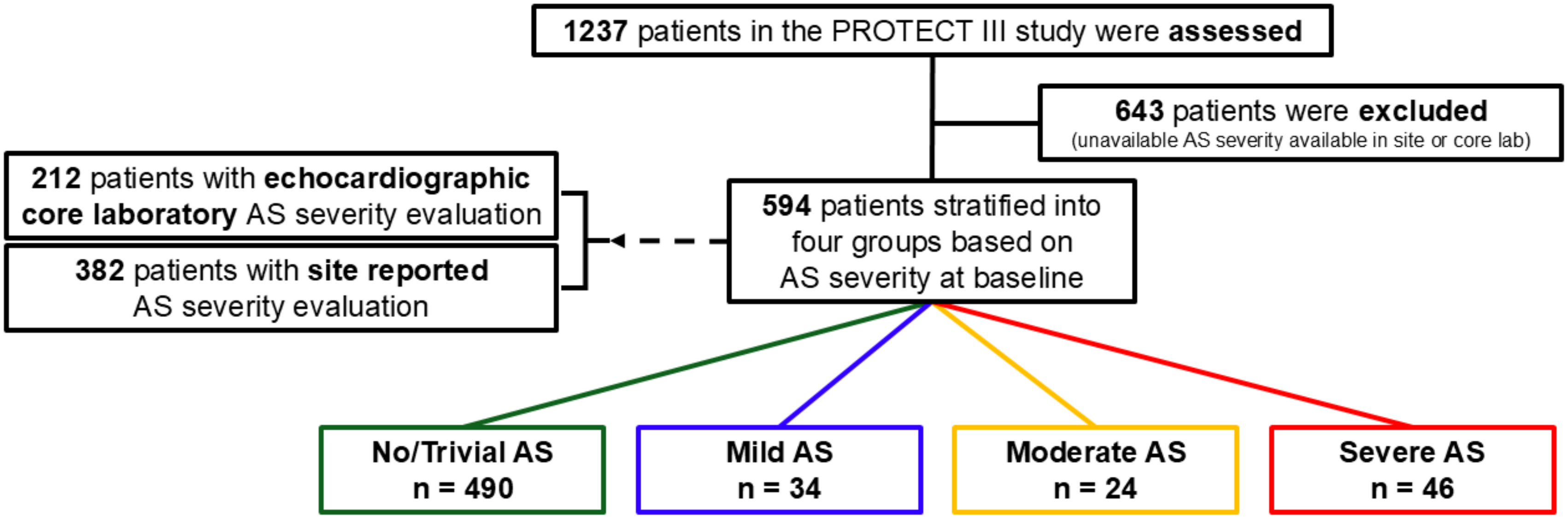

Supplemental Figure S2: Major adverse cardiovascular and cerebrovascular events at 30 and 90-days was similar between patients with severe aortic stenosis and not-severe aortic stenosis

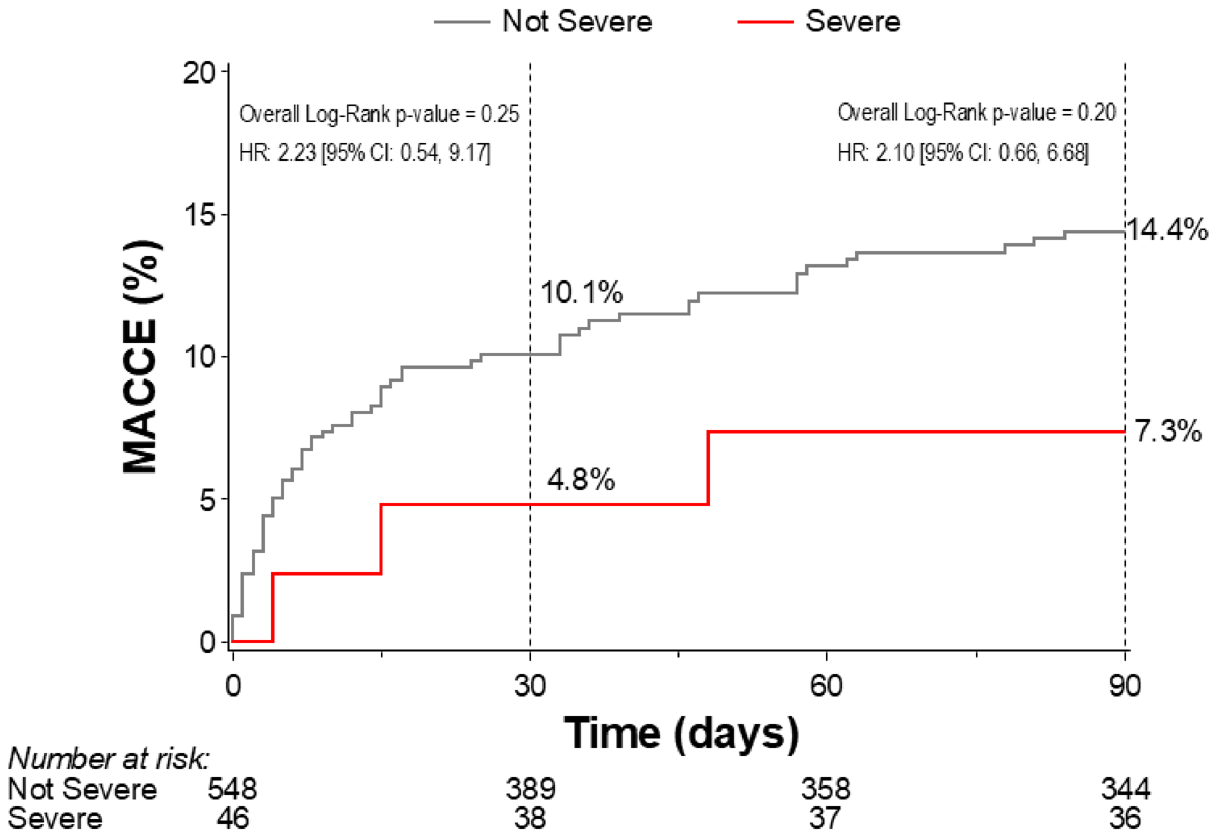

MACCE denotes major adverse cardiovascular and cerebrovascular events.

Supplemental Figure S3: All-Cause Death at 1-year was similar between patients with severe aortic stenosis and not-severe aortic stenosis

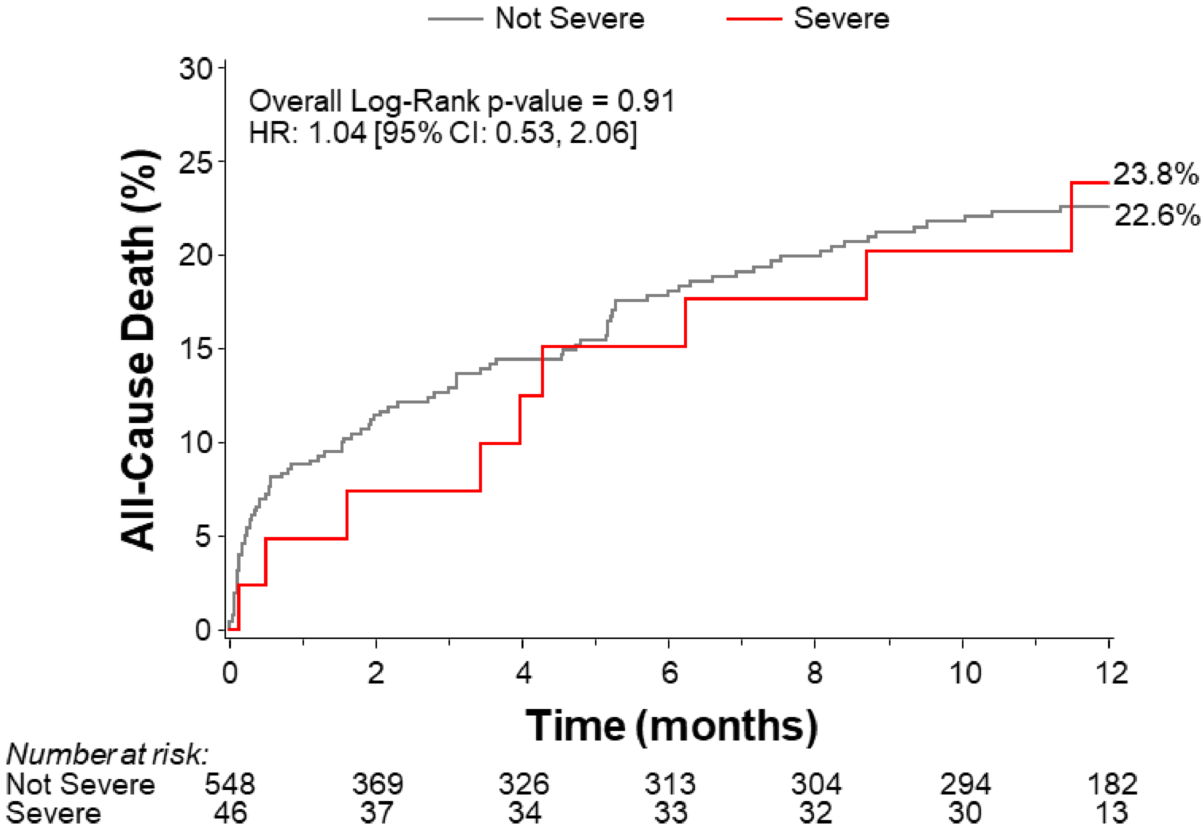

Supplemental Table S1: Characteristics of patients included and excluded

	Included (N=594)	Excluded (N=643)	P-value
Demographics			
Age, years	71.3 ± 11.3	70.7 ± 10.9	0.37
Sex, male	71.7% (426/594)	74.5% (479/643)	0.27
Race			
White/Caucasian	69.2% (411/594)	65.3% (420/643)	0.15
Black/African American	10.9% (65/594)	13.7% (88/643)	0.14
Asian	3.5% (21/594)	2.6% (17/643)	0.36
American Indian or Alaska Native	0.5% (3/594)	0.5% (3/643)	0.92
Native Hawaiian/Pacific Islander	0.2% (1/594)	0% (0/643)	0.30
Other	3.9% (23/594)	3.0% (19/643)	0.37
Unknown race	11.8% (70/594)	14.9% (96/643)	0.10
Body mass index, kg/m^2	28.5 ± 6.4	28.8 ± 6.5	0.57
Medical History			
HTN	89.3% (525/588)	94.2% (603/640)	0.002
Dyslipidemia	77.1% (450/584)	82.6% (527/638)	0.02
History of tobacco use	63.3% (365/577)	61.1% (384/628)	0.45
Diabetes mellitus	57.7% (339/588)	54.4% (349/641)	0.26
Anemia	18.2% (97/532)	21.2% (117/551)	0.21
PVD	19.7% (115/583)	24.4% (155/634)	0.05
Chronic pulmonary disease	23.2% (135/581)	22.0% (140/636)	0.61
Prior stroke/TIA	17.6% (103/584)	16.6% (106/638)	0.64
CKD	29.6% (172/582)	33.9% (217/641)	0.11
eGFR[§], mL/min/1.73 m^2	68.8 ± 24.6	67.3 ± 24.5	0.37
On dialysis	32.0% (55/172)	25.8% (56/217)	0.18
Prior MI	37.0% (210/567)	43.5% (269/618)	0.02
CAD	78.8% (460/584)	87.2% (558/640)	<0.0001
Prior PCI	33.8% (198/586)	42.8% (270/631)	0.001
Prior CABG	10.9% (64/589)	18.2% (116/639)	0.0003
Angina	39.7% (215/542)	47.4% (267/563)	0.009
CHF	0.7% (4/544)	0.5% (3/569)	0.66
Prior pacemaker/ICD/CRT implantation	15.6% (87/558)	18.7% (109/584)	0.17
AF	34.8% (16/46)	38.2% (26/68)	0.71
Admission Characteristics			
ACS as primary reason for admission	61.2% (324/529)	52.3% (290/555)	0.003
Echocardiography Characteristics			
VHD	12.6% (75/594)	7.4% (8/108)	0.12
LVEF, %	35.3 ± 15.6	32.5 ± 14.7	0.007
Angiography Characteristics			
LM disease	60.4% (357/591)	56.9% (362/636)	0.22
Number of diseased vessels			0.02
1	8.4% (49/585)	13.9% (89/641)	0.002
2	30.4% (178/585)	30.9% (198/641)	0.86
3	59.5% (348/585)	53.7% (344/641)	0.04
>3	1.7% (10/585)	1.6% (10/641)	0.84
Number of vessels treated			0.95
1	28.4% (159/559)	29.2% (171/585)	0.77
2	45.1% (252/559)	44.3% (259/585)	0.78
3	26.5% (148/559)	26.5% (155/585)	0.99
Clinical Outcomes			
90-day MACCE	13.8% (70)	11.5% (60)	0.25
1-year All-Cause Mortality	22.7% (105)	18.2% (86)	0.08

Supplemental Table S2: Additional Echocardiographic Features Stratified by Severity of Aortic Stenosis

	No or trace AS (N=490)	Mild AS (N=34)	Moderate AS (N=24)	Severe AS (N=46)	P value*
**Left ventricular function**					
Normal	17.2% (75/437)	32.1% (9/28)	23.8% (5/21)	38.1% (16/42)	0.003
Mild dysfunction	9.8% (43/437)	7.1% (2/28)	14.3% (3/21)	9.5% (4/42)	0.87
Moderate dysfunction	18.3% (80/437)	17.9% (5/28)	14.3% (3/21)	16.7% (7/42)	0.96
Severe dysfunction	54.7% (239/437)	42.9% (12/28)	47.6% (10/21)	35.7% (15/42)	0.08
**Right ventricular function**					
Normal	70.4% (276/392)	69.2% (18/26)	73.7% (14/19)	78.4% (29/37)	0.76
Mild dysfunction	17.6% (69/392)	15.4% (4/26)	15.8% (3/19)	10.8% (4/37)	0.76
Moderate dysfunction	9.4% (37/392)	15.4% (4/26)	5.3% (1/19)	10.8% (4/37)	0.69
Severe dysfunction	2.6% (10/392)	0% (0/26)	5.3% (1/19)	0% (0/37)	0.51
**Mitral valve stenosis**					
No/Trace	95.1% (369/388)	78.3% (18/23)	66.7% (12/18)	80.6% (25/31)	<0.0001
Mild	4.4% (17/388)	17.4% (4/23)	16.7% (3/18)	6.5% (2/31)	0.01
Moderate	0.5% (2/388)	4.3% (1/23)	16.7% (3/18)	12.9% (4/31)	<0.0001
Severe[§]	0% (0/388)	0% (0/23)	0% (0/18)	0% (0/31)	N/A
**Aortic valve regurgitation**					
No/Trace	76.1% (360/473)	60% (18/30)	54.5% (12/22)	50% (19/38)	0.0003
Mild	19.7% (93/473)	36.7% (11/30)	27.3% (6/22)	28.9% (11/38)	0.08
Moderate	4.2% (20/473)	3.3% (1/30)	18.2% (4/22)	18.4% (7/38)	0.0002
Severe[§]	0% (0/473)	0% (0/30)	0% (0/22)	2.6% (1/38)	0.003
**Tricuspid valve regurgitation**					
Absent	26.4% (53/201)	50% (4/8)	0% (0/3)	0% (0/5)	0.16
Mild	56.7% (114/201)	50% (4/8)	33.3% (1/3)	60% (3/5)	0.85
Moderate	14.4% (29/201)	0% (0/8)	33.3% (1/3)	40% (2/5)	0.19
Severe[§]	2.5% (5/201)	0% (0/8)	33.3% (1/3)	0% (0/5)	0.01

Values are mean (± standard deviation) or % (n/N). *Refers to overall p-value. [§]patients with severe valvular lesions were excluded from the current study. AS denotes aortic stenosis.

Supplemental Table S3. Additional Angiographic Characteristics Stratified by Severity of Aortic Stenosis

	No or trace AS (N=490)	Mild AS (N=34)	Moderate AS (N=24)	Severe AS (N=46)	P value*
**Pre-PCI SYNTAX Score**	28.8 ± 12.8	31.0 ± 9.2	32.6 ± 15.6	21.8 ± 10.5	0.009
**Pre-PCI Ischemia Jeopardy Score**	9.0 ± 2.1	9.8 ± 1.3	9.1 ± 2.3	8.5 ± 2.4	0.10
**Number of lesions treated per patient**	2.0 [2.0, 3.0]	2.0 [1.0, 3.0]	2.0 [1.0, 3.0]	2.0 [1.0, 3.0]	0.55
**Vessel location**[§]					
LM	46.1% (225/488)	61.8% (21/34)	50% (12/24)	58.7% (27/46)	0.14
LAD	75.8% (370/488)	73.5% (25/34)	54.2% (13/24)	67.4% (31/46)	0.08
LCX	57.0% (278/488)	55.9% (19/34)	54.2% (13/24)	32.6% (15/46)	0.02
RCA	33.2% (162/488)	26.5% (9/34)	25% (6/24)	23.9% (11/46)	0.44
Grafts	3.7% (18/488)	5.9% (2/34)	0% (0/24)	0% (0/46)	0.36
**Lesion Location**[§]					
Proximal	78.2% (372/476)	58.8% (20/34)	66.7% (16/24)	54.3% (25/46)	0.0004
Middle	58.0% (276/476)	55.9% (19/34)	54.2% (13/24)	50% (23/46)	0.75
Distal	38.2% (182/476)	35.3% (12/34)	20.8% (5/24)	30.4% (14/46)	0.27
Ostial	20.4% (97/476)	32.4% (11/34)	25% (6/24)	28.3% (13/46)	0.26
Lesion Length (mm)	10.9 [5.8, 19.9]	7.0 [3.7, 15.6]	11.7 [4.7, 20.9]	8.8 [5.0, 16.0]	0.005
**Calcification**[§]					
None/Mild	32.2% (354/1099)	41.3% (33/80)	17.8% (8/45)	27.9% (24/86)	0.04
Moderate	16.7% (183/1099)	8.8% (7/80)	11.1% (5/45)	12.8% (11/86)	0.20
Severe	51.1% (562/1099)	50% (40/80)	71.1% (32/45)	59.3% (51/86)	0.03
**Pre-PCI TIMI**[§]					
0	10.9% (45/413)	17.2% (5/29)	0% (0/19)	2.7% (1/37)	0.10
1	3.1% (13/413)	0% (0/29)	5.3% (1/19)	0% (0/37)	0.48
2	3.4% (14/413)	0% (0/29)	0% (0/19)	0% (0/37)	0.40
3	82.6% (341/413)	82.8% (24/29)	94.7% (18/19)	97.3% (36/37)	0.07
**Post-PCI TIMI**[§]					
0	0.7% (3/417)	0% (0/29)	0% (0/20)	0% (0/37)	0.89
1	0.5% (2/417)	0% (0/29)	0% (0/20)	0% (0/37)	0.94
2	0.7% (3/417)	0% (0/29)	0% (0/20)	2.7% (1/37)	0.55
3	98.1% (409/417)	100% (29/29)	100% (20/20)	97.3% (36/37)	0.78
**Residual SYNTAX Score**	7.5 ± 8.8	5.1 ± 6.6	6.8 ± 9.8	3.4 ± 4.3	0.046
**Change in SYNTAX Score**	-21.4 ± 11.0	-26.0 ± 8.6	-25.8 ± 14.6	-18.5 ± 9.2	0.03
**Residual Ischemia Jeopardy Score**	2.1 ± 2.2	1.5 ± 1.7	2.0 ± 2.7	1.1 ± 1.4	0.02
**Change in Ischemia Jeopardy Score**	-6.9 ± 2.5	-8.3 ± 1.8	-7.1 ± 2.8	-7.4 ± 2.3	0.02

Values are mean (± standard deviation), median [Q1, Q3] or % (n/N). *refers to overall p-value; [§] Lesion-based analysis. AS denotes aortic stenosis; PCI, percutaneous coronary; SYNTAX, the Synergy between PCI with Taxus and Cardiac Surgery. LM, left main artery; LAD, left anterior descending artery; LCX, left circumflex artery; RCA, right coronary artery; TIMI, thrombolysis in myocardial infarction.

Supplemental Table S4. Procedural Characteristics Stratified by Severity of Aortic Stenosis

	No or trace AS (N=490)	Mild AS (N=34)	Moderate AS (N=24)	Severe AS (N=46)	P value*
**Duration of index PCI, hours**	1.8 [1.2, 2.7]	1.9 [1.3, 2.7]	2.5 [1.8, 3.3]	2.0 [1.3, 2.6]	0.08
**Contrast volume, mL**	207.1 ± 104.3	195.9 ± 120.4	220.4 ± 150.0	208.1 ± 111.5	0.87
**PCI access**					
Radial	18.8% (87/462)	16.7% (5/30)	4.3% (1/23)	6.5% (3/46)	0.06
Brachial	0.2% (1/462)	0% (0/30)	0% (0/23)	0% (0/46)	0.98
Femoral	79.7% (368/462)	80% (24/30)	95.7% (22/23)	93.5% (43/46)	0.04
Other	1.3% (6/462)	3.3% (1/30)	0% (0/23)	0% (0/46)	0.58
**Atherectomy use**	37.1% (179/483)	51.5% (17/33)	75.0% (18/24)	54.3% (25/46)	0.0002
**Intravascular imaging use (IVUS/OCT)**	43.7% (211/483)	42.4% (14/33)	62.5% (15/24)	58.7% (27/46)	0.08
**Impella device used**					
Impella 2.5	33.3% (163/490)	29.4% (10/34)	4.2% (1/24)	19.6% (9/46)	0.007
Impella CP	66.7% (327/490)	70.6% (24/34)	95.8% (23/24)	80.4% (37/46)	0.007
**Successful implant**	99.8% (489/490)	97.1% (33/34)	100% (24/24)	100% (46/46)	0.06
**Impella access**					
Femoral artery	94.9% (465/490)	91.2% (31/34)	95.8% (23/24)	91.3% (42/46)	0.61
Non-femoral (Axillary/Subclavian/Transcaval)	4.9% (24/490)	8.8% (3/34)	4.2% (1/24)	8.7% (4/46)	0.56
**Duration of Impella support**	4.64 ± 11.73	3.80 ± 8.63	13.08 ± 42.07	1.62 ± 1.25	0.01
**Noninvasive hemodynamic measurements before Impella implantation**					
Heart rate, bpm	78.0 ± 18.8	76.4 ± 17.8	70.5 ± 15.6	78.0 ± 17.5	0.30
Mean arterial blood pressure, mmHg	87.3 ± 13.5	89.4 ± 12.8	89.8 ± 13.1	90.3 ± 13.3	0.38
**Noninvasive hemodynamic measurements during Impella support**					
Heart rate, bpm	78.5 ± 18.1	75.7 ± 16.4	70.6 ± 15.0	77.2 ± 16.7	0.25
Mean arterial blood pressure, mmHg	94.7 ± 16.0	95.3 ± 17.9	95.2 ± 14.1	95.1 ± 19.5	1.00
**Stay in ICU during Index hospitalization**					
Total time in ICU (days)	4.0 [2.0, 7.0]	2.0 [1.0, 4.0]	11.0 [8.0, 17.0]	2.8 [2.0, 7.0]	0.003
**Total hospital stay (days)**	8.0 [5.0, 12.0]	8.0 [5.5, 12.0]	7.0 [2.0, 14.0]	7.0 [4.0, 11.0]	0.61

Values are mean ± standard deviation, median [Q1, Q3], or % (n/N). AS denotes aortic stenosis; PCI, percutaneous coronary intervention; IVUS, intravascular ultrasound; OCT, optical coherence tomography; ICU, intensive care unit.
